# Inflammatory bowel disease patients provide reliable self‐reported medical information: A multicentre prospective pharmacovigilance monitoring system

**DOI:** 10.1002/pds.5175

**Published:** 2020-12-01

**Authors:** Pepijn W. A. Thomas, Rachel L. West, Maurice G. V. M. Russel, Jeroen M. Jansen, Leanne J. Kosse, Naomi T. Jessurun, Tessa E. H. Römkens, Frank Hoentjen, IBDREAM registry

**Affiliations:** ^1^ Department of Gastroenterology and Hepatology Radboud University Medical Centre Nijmegen The Netherlands; ^2^ Department of Gastroenterology and Hepatology Franciscus Gasthuis en Vlietland Rotterdam The Netherlands; ^3^ Department of Gastroenterology and Hepatology Medisch Spectrum Twente Enschede The Netherlands; ^4^ Department of Gastroenterology and Hepatology Onze Lieve Vrouwe Gasthuis Amsterdam The Netherlands; ^5^ Netherlands Pharmacovigilance Centre Lareb 's‐Hertogenbosch The Netherlands; ^6^ Department of Gastroenterology and Hepatology Jeroen Bosch Ziekenhuis 's‐Hertogenbosch The Netherlands

**Keywords:** anti‐TNF, biologicals, IBD, patient reporting, PRO

## Abstract

**Purpose:**

To assess the agreement between patient‐reported and health care provider‐reported medical information in inflammatory bowel disease (IBD).

**Methods:**

This multicentre, prospective, event monitoring study enrolled adult Crohn's disease (CD) and ulcerative colitis (UC) patients treated with a biological in four medical centers in the Netherlands. At two‐monthly intervals, patients completed questionnaires on biological use, combination therapy and indication. The patient‐reported information was compared with their electronic health records (EHRs) and analysed for percentage agreement and Cohen's kappa. A reference population from a prospective IBD registry was used to assess the representativeness of the study population.

**Results:**

In total, 182 patients (female 50.5%, mean age 42.2 years, CD 76.9%) were included in the analysis. At baseline, 51.0% of the patients were prescribed an immunomodulator (43.9% thiopurines, 7.1% methotrexate), and patients were prescribed biologicals as follows: 59.3% infliximab, 30.2% adalimumab, 9.3% vedolizumab, and 1.1% ustekinumab. Agreement on patient‐reported indication and biological use was almost perfect (κ = 0.878 and κ = 1.000, respectively); substantial for combination therapy (κ = 0.672). Gender, age, type of IBD, biological use and combination therapy were comparable with the reference population.

**Conclusion:**

Systematic patient‐reporting by questionnaires was reliable in retrieving indication and treatment specific information from IBD patients. These results indicate that the use of patient‐reporting outcomes in daily IBD practice can ensure reliable information collection.

**Key Points**

- This study showed close agreement between patient‐reported biological use and subtype of IBD, and to a lesser extent on IBD specific combination therapy when compared with data from their electronic health records.
- A discrepancy was seen in specific IBD comedication. In these cases patients more often reported steroid use which was not reported by the health care provider (HCP) and HCPs more often reported thiopurine use than patients. This suggests that the use of prescribed drugs should more often be discussed and documented.
- This online reporting tool may be used in clinical practice to identify patients who are not taking their medication as prescribed by their HCP.
- Patients provide reliable medical information that can be used for patient‐reported adverse drug reactions (ADRs) to more accurately evaluate the ADR.

## 1 INTRODUCTION

Patient‐reported outcomes (PROs) provide a tool to monitor and optimize treatment of inflammatory bowel disease (IBD), a chronic bowel condition with a remitting and relapsing disease course. In order to draw reliable clinical conclusions from PROs, the accuracy and reliability of this information has to be assessed. In the last decades, biologic agents have emerged as effective therapy in IBD treatment^1^ but are also associated with adverse drug reactions (ADRs).^2^ Post‐marketing safety surveillance depends on reporting by health care providers (HCPs), but ADRs are under‐reported.^3^ In order to accurately evaluate the real‐world ADRs, reliable patient‐reported information about treatment and disease is needed. Two previous studies have reported close agreement between patient and HCP for self‐reported subtype of IBD (Crohn's disease, CD or ulcerative colitis, UC), but these studies did not address the use of IBD medication.^4^, ^5^

The Dutch Pharmacovigilance Centre has developed a web‐based tool ‐ *Dutch Biologic Monitor* ^6^ ‐ for patients with immunological mediated immune diseases to report ADRs related to biologic agents. We used this tool to evaluate the agreement between IBD patient‐reported and HCP‐reported subtype of IBD and IBD medication use.

## 2 METHODS

### 2.1 Design

This prospective multicentre event monitoring study evaluated the quality of patient‐reported information on IBD subtype and IBD drug use during biological treatment, using the *Dutch Biologic Monitor*.^6^ The study was conducted in four medical centers in the Netherlands between January 1, 2017 and December 31, 2018. The study protocol was approved by the ethics committee [NW2016‐66] (METC Brabant).

### 2.2 Cohorts

We used two cohorts. The first cohort consisted of IBD patients enrolled in the *Dutch Biologic Monitor* and was analysed for agreement between patient‐reported medical information and electronic health records (EHRs). The second cohort consisted of IBD patients enrolled in IBDREAM,^7^ a multicentre prospective IBD registry used in all four participating hospitals, and was used to analyse the representativeness of the first cohort.

#### 2.2.1 Cohort 1: Study population

Patients ≥18 years of age with a diagnosis of CD or UC were eligible, provided they were being treated with a biological. Patients were recruited consecutively during outpatient visits, via letters from the outpatient pharmacy, or during infusion therapy. All participants signed a web‐based informed consent. At baseline, participants in the *Dutch Biologic Monitor* completed a comprehensive web‐based questionnaire, which included demographic data, IBD drug use, indication for biological therapy, co‐morbidities and ADRs. Indication of therapy, biological therapy and combination therapy were predefined options (Table S1). Follow‐up questionnaires were completed at an interval of 2 months on drug use and ADRs. Subsequent questionnaires were not sent if the previous questionnaire had expired (no response within 21 days of receiving the questionnaire).

#### 2.2.2 Cohort 2: Reference population

Patients ≥18 years of age with CD or UC diagnosis were included provided they were using a biological at median completion date of the questionnaires on 19 October 2017. Demographics (age and gender), disease type (CD or UC) and IBD medical treatment (biologicals, immunomodulator, prednisone and mesalamine) were retrieved from IBDREAM.^7^

### 2.3 Comparing patient‐ and clinician‐reported medical information

Patient‐reported data were compared with their documented EHR on type of biological therapy, and combination treatment with immunosuppressive agents. Patient‐reported data from the baseline questionnaire were compared with EHR data prior to completion date of the baseline questionnaire. Agreement was defined as a complete match of patient‐ and HCP‐reported information. If patients had not filled in an answer on the use of comedication, no agreement was met.

### 2.4 Statistical analysis

Demographics and baseline characteristics were reported as median with interquartile range or number with proportions. Differences between study population and reference population were assessed using Chi‐Square or students T‐test when appropriate. Fisher's exact test with Monte Carlo simulation was used for differences in biological use and combination therapy. Cohen's kappa and percentage agreement were used to determine agreement between patient‐ and HCP‐reported information for indication, biological generic and brand name, and combination therapy.^8^ Cohen's kappa was interpreted on predefined scores.^8^ Data analysis was performed with SPSS 25.0 (SPSS Inc.; Chicago, IL, USA). *p*‐values <0.05 were considered statistically significant.

## 3 RESULTS

In total, 193 IBD patients were enrolled in the *Dutch Biologic Monitor*. Nine patients were excluded because they did not provide informed consent, two patients discontinued the biological prior to the baseline questionnaire completion date, and one patient was under the age of 18 years (Figure S1). The reference population retrieved from the IBDREAM registry comprised 878 patients. Baseline characteristics of the study population and reference population are presented in Table 1.

**TABLE 1:** Baseline characteristics of the study and reference population

	Study population	Reference population	
	(*n* = 182)	(*n* = 878)	*p*‐value
Female, *n* (%)	92 (50.5)	511 (58.2)	0.059
Age in years, mean ± SD	42.2 ± 14.2	41.5 ± 14.9	0.608
Indication, *n (%)*			0.303
Crohn's disease	140 (76.9)	705 (80.3)	
Ulcerative Colitis	42 (23.1)	173 (19.7)	
Biological prescriptions, *n (%)*			0.472
Adalimumab	55 (30.2)	281 (32.0)	
Golimumab	0 (0)	2 (0.2)	
Infliximab	108 (59.3)	490 (55.8)	
Ustekinumab	2 (1.1)	30 (3.4)	
Vedolizumab	17 (9.3)	75 (8.5)	
Combination therapy, *n (%)*			0.548
Mesalamine	19 (10.4)	116 (13.2)	
Azathioprine	39 (21.4)	152 (17.3)	
Mercaptopurine	26 (14.3)	98 (11.2)	
Thioguanine	15 (8.2)	69 (7.9)	
Methotrexate	13 (7.1)	42 (4.8)	
Corticosteroids	8 (4.4)	47 (5.4)	
Sulfasalazine	2 (1.1)	6 (0.7)	
None	74 (40.7)	426 (48.5)	
IBD medication use^a^			0.163
1	74 (40.7)	426 (48.5)	
2	94 (51.6)	376 (42.8)	
3	14 (7.7)	74 (8.4)	
4	0 (0)	2 (0.2)	

*Note: p*‐values were calculated for the difference between the study and reference population. The indication, biological and combination therapy presented are data derived from the electronic health record.

Abbreviations: IBD, inflammatory bowel disease; IQR, interquartile range; SD, standard deviation.

^a^ Medication count was defined as follows: 1 = biological; 2 = biological and one combination therapy; 3 = biological and two combination therapies; 4 = biological and three combination therapies.

### 3.1 Agreement patient‐reported and HCP‐reported medical information

**FIGURE 1:**
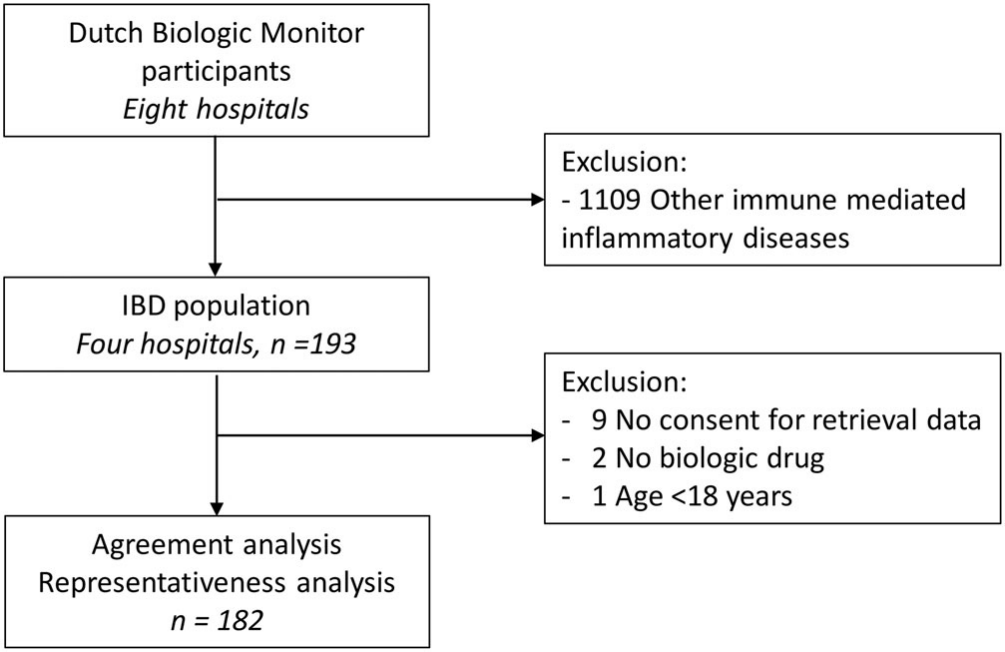
Agreement between patient‐reported and clinician‐reported information in electronic health records. κ, level of interrater agreement

The level of agreement between patient‐ and HCP‐reported information was assessed for 182 IBD patients. Information from baseline questionnaires was compared with patients' EHR (Figure 1). Agreement on treatment indication (95.6%, κ = 0.878), biological generic name (100%, κ = 1.000) and biological brand name (90.1%, κ = 0.841) was almost perfect. Three patients who did not meet agreement on indication for biological use, reported both CD and UC. All 18 patients who did not report the same biological brand name as documented in their EHR were on infliximab (e.g. reported Remicade instead of Remsima).

Agreement on combination therapy was substantial (74.2%, κ = 0.672). Five patients (2.7%) had missing data for comedication use and were labeled as no agreement. Agreement on singular IBD therapies was as follows: mesalamine (95.1%, κ = 0.773); thiopurine (91.8%, κ = 0.773); prednisone (89.5%, κ = 0.300); and methotrexate (98.4%, κ = 0.861). If agreement was not met, patients more often reported corticosteroid use than was stated in their EHR (16 (8.8%) versus 3 (2.7%), respectively). In contrast, thiopurine use was more often not reported by patients than was stated in their EHR (5 (2.7%) versus 10 (5.5%), respectively). Agreement for combination therapy tended to be lower in patients aged 60 years and older (Table S2). No gender differences were found (Table S3).

### 3.2 Treatment adjustments

Agreement on change in biological therapy was assessed in patients who completed at least one follow‐up questionnaire (*n* = 124). Median follow‐up in these patients was 16 months (IQR 9.5–16). Treatment adjustments were reported by 18 patients (14.5%), while 33 (26.6%) were reported in their EHR. Change in biological brand was reported only once (1/16), whereas temporary or permanent cessation or switch to another biological was reported adequately by all patients (17/17). One patient incorrectly reported that the biological was discontinued (Table S4).

### 3.3 Reference population

Study population (*Dutch Biologic Monitor*) and reference population (IBDREAM registry) were similar in gender, mean age, indication, biological and combination therapy, as presented in Table 1.

## 4 DISCUSSION

This study showed close agreement between patient‐reported information and their EHR on disease subtype and biological use, and to a lesser extent, on IBD‐specific combination therapy. Agreement on reported combination therapy was lower in patients aged ≥60 years. Change in therapy was reported adequately in case of cessation or switch to another biological, but to a lesser extent when the change was to another biological brand name.

Our findings are consistent with two previous studies that showed good reliability of self‐reported diagnosis in IBD patients compared with either EHR or another database.^4^, ^5^ Furthermore, our findings of perfect agreement between self‐report and EHR indicate that patients provide reliable information on their drug use. These results were similar to those of a large rheumatology cohort study that used the same methodology for reporting treatment indication and generic biological use (κ = 0.832 and 96% agreement, respectively).^6^ In addition, all clinically relevant biological changes were reported by patients. However, this reporting tool does not seem to reliably detect use specific biological brands (biosimilars). One out six patients using infliximab did not correctly report the infliximab brand name. This should be taken into account when using patient‐reports for the safety and effectiveness assessment of specific biosimilars.

Agreement on IBD specific combination therapy was substantial. Patients more often reported use of IBD combination therapy than was reported in their EHR, especially corticosteroids. This may reflect discrepancies in omission of specific medication reported in the EHR while still being used by the patient.^9^ However, thiopurine use was less often reported by patients, possibly because of suboptimal drug adherence, knowledge of type of drugs or insufficient EHR documentation. Moreover, less agreement was found in patients aged ≥60 years, which may be attributed to polypharmacy being more common in older patients than in younger patients^10^ resulting in poor medication reporting.

### 4.1 Clinical implications

The current under‐reporting of ADRs by HCPs can be improved by using patient‐reporting.^3^, ^11^ Reliable information on therapy indication and drug use is important for an accurate evaluation of the ADR. Our results showed that this information can be directly retrieved from patients and does not necessarily require additional information retrieval from the EHR. Moreover, patient‐reporting may provide valuable insight into the patients' perception and burden of ADRs. An advantage of the questionnaire used in this study is the possibility to share reports directly with pharmacovigilance centers. These reports may help HCPs to identify patients not using prescribed IBD drugs correctly and to improve therapy adherence.^12^ Since ADRs and medication adherence are inversely related, early recognition and discussion of ADRs could improve adherence.^13^ Finally, this reporting system may reveal new as yet unknown ADRs that HCPs are unaware of. Early detection could prevent potential harm by timely treatment adjustments.

### 4.2 Limitations and strengths

This multicentre prospective study included a large number of participants and provided extensive patient‐reported information. However, as patients received an open invitation to report on ADRs, this may have resulted in participation bias. Moreover, 89% of the patients used a first line biological (infliximab or adalimumab), and patient‐reported information may be less reliable in patients receiving a second, third or in the future, a fourth line biological.

## 5 CONCLUSION

This reported method of online self‐reporting is reliable for directly retrieving disease and treatment specific information from IBD patients on biological therapy. This reporting system may contribute to improving medication adherence and provides valuable information for future use of patient‐reporting in the context of drug safety.

## ETHICS STATEMENT

The study protocol was approved by the ethics committee [NW2016‐66] (METC Brabant), and by the local ethics committees from the participating hospitals.

## CONFLICT OF INTEREST

The authors have no conflicts of interest to declare.

## Supporting information


**Figure S1.** Flowchart study design. The Dutch Biologic Monitor included several immune mediated inflammatory disease. Data from inflammatory bowel disease (IBD) patients originated from four hospitals. The study population comprised of the participants that completed the baseline questionnaire. Agreement between patient and clinician reported medical information was assessed, and the representativeness between a reference population and study population.

**Table S1.** Predefined checkbox options for biologicals, indication for biological therapy and combination therapy.
**Table S2.** Differences in agreement on combination therapy between patient‐reported information and clinician reported information in electronic health records for different age groups.
**Table S3.** Differences in agreement on combination therapy between patient‐reported information and clinician reported information in electronic health records for males and females.
**Table S4.** Treatment adjustments reported by patients and in their electronic health records.
