# Supplementary figures and images for: Inflammatory bowel disease patients provide reliable self‐reported medical information: A multicentre prospective pharmacovigilance monitoring system
Source: Pharmacoepidemiol Drug Saf. 2020 Dec 1;30(4):520–4. doi: 10.1002/pds.5175 (PMC7983909; doi:10.1002/pds.5175)

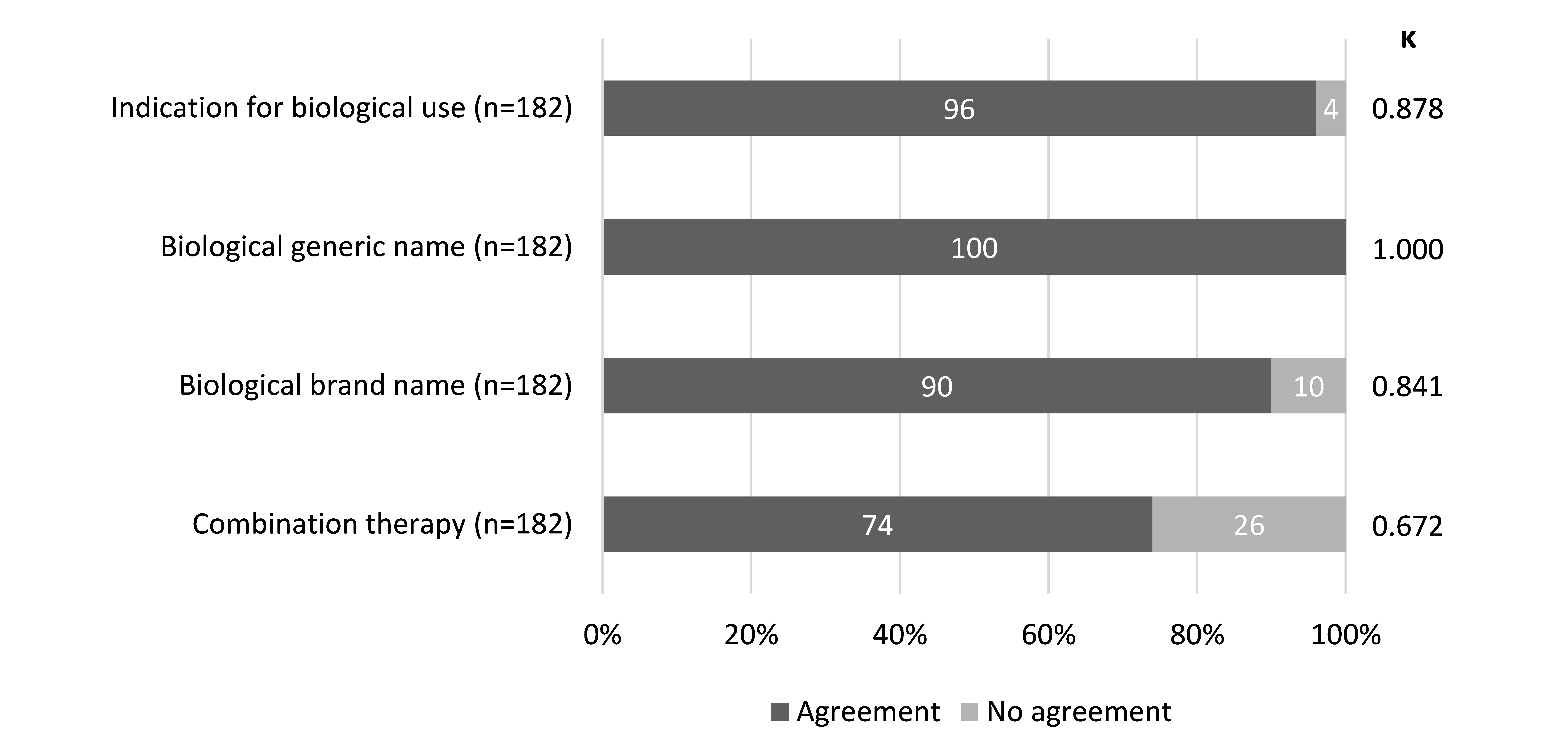

Supplement: Supplementary file 1 — Figure S1. Flowchart study design. The Dutch Biologic Monitor included several immune mediated inflammatory disease. Data from inflammatory bowel disease (IBD) patients originated from four hospitals. The study population comprised of the participants that completed the baseline questionnaire. Agreement between patient and clinician reported medical information was assessed, and the representativeness between a reference population and study population. [file PDS-30-520-s002.tif]
