# Supplementary material for: Inflammatory bowel disease patients provide reliable self‐reported medical information: A multicentre prospective pharmacovigilance monitoring system
Source: Pharmacoepidemiol Drug Saf. 2020 Dec 1;30(4):520–4. doi: 10.1002/pds.5175 (PMC7983909; doi:10.1002/pds.5175)
Supplement: Supplementary file 2 — Table S1. Predefined checkbox options for biologicals, indication for biological therapy and combination therapy. Table S2. Differences in agreement on combination therapy between patient‐reported information and clinician reported information in electronic health records for different age groups. Table S3. Differences in agreement on combination therapy between patient‐reported information and clinician reported information in electronic health records for males and females. Table S4. Treatment adjustments reported by patients and in their electronic health records. [file PDS-30-520-s001.docx]

**Appendix**

**SUPPLEMENTARY Figure 1.** Flowchart study design. The Dutch Biologic Monitor included several immune mediated inflammatory disease. Data from inflammatory bowel disease (IBD) patients originated from four hospitals. The study population comprised of the participants that completed the baseline questionnaire. Agreement between patient and clinician reported medical information was assessed, and the representativeness between a reference population and study population.

| **SUPPLEMENTARY Table 1:** Predefined checkbox options for biologicals, indication for biological therapy and combination therapy. | | |
| --- | --- | --- |
| **Biological** | **Indication** | **Combination therapy** |
| Amgevita® (adalimumab) | Crohn’s disease | Azathioprine |
| Cyltezo® (adalimumab) | Ulcerative colitis | Hydrocortisone |
| Halimatoz® (adalimumab) |  | Mercaptopurine |
| Hefiya® (adalimumab) |  | Mesalamine |
| Hulio® (adalimumab) |  | Methotrexate |
| Humira® (adalimumab) |  | Methylprednisolone |
| Idacio® (adalimumab) |  | Prednisone/Prednisolone |
| Imraldi® (adalimumab) |  | Sulfasalazine |
| Kromeya® (adalimumab) |  | 6-Thioguanine |
| Solymbic® (adalimumab) |  |  |
| Simponi® (golimumab) |  |  |
| Flixabi® (infliximab) |  |  |
| Inflectra® (infliximab) |  |  |
| Remicade® (infliximab) |  |  |
| Remsima® (infliximab) |  |  |
| Zessly® (infliximab) |  |  |
| Stelara® (ustekinumab) |  |  |
| Entyvio® (vedolizumab) |  |  |
| *All biologicals and combination therapy listed in this table apply for the treatment of Crohn’s disease and ulcerative colitis, except for Stelara® (ustekinumab) which was only registered for the treatment of Crohn’s disease during this study.* | | |

*Differences in agreement on combination therapy for age groups and gender.*

| **Supplementary Table 2:** Differences in agreement on combination therapy between patient-reported information and clinician reported information in electronic health records for different age groups. | | | | |
| --- | --- | --- | --- | --- |
|  |  | **Age Group** | |  |
|  |  | **18-59 years**  *n (%*) | **60 years or older**  *n (%)* | **p-value** |
| **Agreement** | Yes | 122 (76) | 13 (62) | 0.189 |
|  | No | 39 (24) | 8 (38) |  |
|  | Total | 161 (100) | 21 (100) |  |

| **Supplementary Table 3:** Differences in agreement on combination therapy between patient-reported information and clinician reported information in electronic health records for males and females. | | | | |
| --- | --- | --- | --- | --- |
|  |  | **Gender** | |  |
|  |  | **Male**  *n (%)* | **Female**  *n (%)* | **p-value** |
| **Agreement** | Yes | 64 (71) | 71 (77) | 0.399 |
|  | No | 26 (29) | 21 (23) |  |
|  | Total | 90 (100) | 92 (100) |  |

| **SUPPLEMENTARY Table 4: Treatment adjustments reported by patients and in their electronic health records.** | | | |
| --- | --- | --- | --- |
|  | **Patient reported change**  *n* | **Patient reported no change**  *n* | **Level of interrater agreement**  Level (κ) |
| EHR documented change, *n* | 17 | 16 |  |
| EHR documented no change, *n* | 1 | 91 |  |
| Agreement* | 107 | 18 | 0.590 |
| EHR = Electronic Health Record  Agreement on reasons for change: switch to biosimilar (n=4), insufficient response (n=7), remission (n=1), pregnancy (n=3), adverse events (n=2).  If changes were only documented in the EHR: switch to biosimilar (n=15), remission (n=1).  If change was only reported by patient: discontinuation treatment, no reason documented (n=1) | | | |
